# Supplementary material for: Hawk Tea Flavonoids as Natural Hepatoprotective Agents Alleviate Acute Liver Damage by Reshaping the Intestinal Microbiota and Modulating the Nrf2 and NF-κB Signaling Pathways
Source: Nutrients. 2022 Sep 5;14(17):3662. doi: 10.3390/nu14173662 (PMC9459715; doi:10.3390/nu14173662)
Supplement: Supplementary file 1 [file nutrients-14-03662-s001.zip › nutrients-1875575-supple/table S1.pdf]

Table S1. The list of total 191 metabolites identified in the hawk tea flavonoids								
**Number**	**Class I**	**Class II**	**CAS**	**Compounds**	**Content**	**Content(%)**	**OB**	**DL**
1	Flavonoids	Isoflavones	52250-35-8	2',7-Dihydroxy-3',4'-dimethoxyisoflavan	1.44E+06	0.23	4.56	0.27
2	Flavonoids	Isoflavones	1156-78-1	2'-Hydroxygenistein	1.25E+05	0.02	ND	ND
3	Flavonoids	Isoflavones	51011-05-3	6"-O-Malonylgenistin	2.96E+04	0.00	ND	ND
4	Flavonoids	Isoflavones	-	Genistein-7-O-galactoside-rhamnose	3.61E+05	0.06	ND	ND
5	Flavonoids	Isoflavones	66026-80-0	Genistein-8-C-glucoside	2.43E+07	3.88	15.27	0.72
6	Flavonoids	Flavonols	1486-70-0	3-O-Methylquercetin	9.74E+04	0.02	10.1	0.3
7	Flavonoids	Flavonols	-	6-Hydroxykaempferol-7-O-glucoside	1.23E+06	0.20	2.87	0.78
8	Flavonoids	Flavonols	572-30-5	Avicularin(Quercetin-3-O-α-L-arabinofuranoside)	2.17E+07	3.47	2.06	0.7
9	Flavonoids	Flavonols	137225-59-3	Dihydrokaempferide	8.13E+05	0.13	23.1	0.27
10	Flavonoids	Flavonols	-	Dihydrokaempferol-7-O-glucoside	1.16E+06	0.19	ND	ND
11	Flavonoids	Flavonols	1226-22-8	Garbanzol	1.42E+06	0.23	83.67	0.21
12	Flavonoids	Flavonols	-	Herbacetin-3-O-glucuronide	9.87E+06	1.58	ND	ND
13	Flavonoids	Flavonols	480-19-3	Isorhamnetin	1.97E+04	0.00	49.6	0.31
14	Flavonoids	Flavonols	6758-51-6	Isorhamnetin-3,7-O-diglucoside	1.85E+04	0.00	13.44	0.65
15	Flavonoids	Flavonols	-	Isorhamnetin-3-O-(6"-acetylglucoside)	3.32E+05	0.05	ND	ND
16	Flavonoids	Flavonols	5041-82-7	Isorhamnetin-3-O-Glucoside	4.36E+05	0.07	1.17	0.8
17	Flavonoids	Flavonols	55033-90-4	Isorhamnetin-3-O-neohesperidoside	7.92E+04	0.01	ND	ND
18	Flavonoids	Flavonols	-	Isorhamnetin-3-O-rhamnoside	4.70E+04	0.01	ND	ND
19	Flavonoids	Flavonols	520-18-3	Kaempferol (3,5,7,4'-	8.84E+05	0.14	41.88	0.24

				Tetrahydroxyflavone)				
20	Flavonoids	Flavonols	482-38-2	Kaempferol-3,7-O-dirhamnoside (Kaempferitrin)	2.88E+06	0.46	8.16	0.79
21	Flavonoids	Flavonols	-	Kaempferol-3-O-(2"-acetyl)glucoside	1.33E+07	2.12	ND	ND
22	Flavonoids	Flavonols	-	Kaempferol-3-O-(4"-p-coumaroyl)rhamnoside	3.68E+05	0.06	ND	ND
23	Flavonoids	Flavonols	-	Kaempferol-3-O-(6"-malonyl)galactoside	1.16E+06	0.19	ND	ND
24	Flavonoids	Flavonols	-	Kaempferol-3-O-(6"-malonyl)glucoside	5.87E+04	0.01	ND	ND
25	Flavonoids	Flavonols	20316-62-5	Kaempferol-3-O-(6"-p-Coumaroyl)glucoside (Tiliroside)	4.47E+06	0.71	1.94	0.66
26	Flavonoids	Flavonols	5041-67-8	Kaempferol-3-O-arabinoside (Juglanin)	1.33E+06	0.21	2.73	0.65
27	Flavonoids	Flavonols	23627-87-4	Kaempferol-3-O-galactoside (Trifolin)	1.28E+07	2.05	19.61	0.74
28	Flavonoids	Flavonols	480-10-4	Kaempferol-3-O-glucoside (Astragalin)	2.91E+06	0.47	2.77	0.74
29	Flavonoids	Flavonols	-	Kaempferol-3-O-glucoside-7-O-rhamnoside	4.61E+06	0.74	ND	ND
30	Flavonoids	Flavonols	22688-78-4	Kaempferol-3-O-glucuronide	2.38E+06	0.38	2.65	0.76
31	Flavonoids	Flavonols	32602-81-6	Kaempferol-3-O-neohesperidoside	4.23E+06	0.68	5.78	0.75
32	Flavonoids	Flavonols	482-39-3	Kaempferol-3-O-rhamnoside (Afzelin)(Kaempferin)	2.62E+06	0.42	3.83	0.7
33	Flavonoids	Flavonols	16290-07-6	Kaempferol-7-O-glucoside	1.39E+07	2.21	2.8	0.68
34	Flavonoids	Flavonols	20196-89-8	Kaempferol-7-O-rhamnoside	2.43E+06	0.39	2.27	0.72
35	Flavonoids	Flavonols	-	Morin-3-O-xyloside	2.98E+07	4.76	ND	ND
36	Flavonoids	Flavonols	529-44-2	Myricetin	2.93E+05	0.05	13.75	0.31
37	Flavonoids	Flavonols	-	Myricetin-3-O-(6"-acetyl)glucoside	3.07E+04	0.00	ND	ND
38	Flavonoids	Flavonols	-	Myricetin-3-O-(6"-malony)glucoside	1.36E+04	0.00	ND	ND

39	Flavonoids	Flavonols	132679-85-7	Myricetin-3-O-arabinoside	9.05E+05	0.14	ND	ND
40	Flavonoids	Flavonols	19833-12-6	Myricetin-3-O-glucoside	8.62E+06	1.38	1.43	0.79
41	Flavonoids	Flavonols	77363-65-6	Myricetin-3-O-glucuronide	8.32E+04	0.01	ND	ND
42	Flavonoids	Flavonols	17912-87-7	Myricetin-3-O-rhamnoside (Myricitrin)	9.72E+04	0.02	5.08	0.77
43	Flavonoids	Flavonols	41093-68-9	Myricetin-3-O-rutinoside	3.16E+06	0.51	ND	ND
44	Flavonoids	Flavonols	117-39-5	Quercetin	3.16E+06	0.51	46.43	0.28
45	Flavonoids	Flavonols	33429-83-3	Quercetin-3,4'-Dimethyl Ether	3.43E+04	0.01	12.7	0.33
46	Flavonoids	Flavonols	28638-13-3	Quercetin-3,7-Di-O-rhamnoside	3.02E+06	0.48	ND	ND
47	Flavonoids	Flavonols	-	Quercetin-3-O-(2''-acetyl)glucuronide	1.84E+05	0.03	ND	ND
48	Flavonoids	Flavonols	-	Quercetin-3-O-(2''-Galloyl)Arabinoside	6.12E+04	0.01	ND	ND
49	Flavonoids	Flavonols	59262-54-3	Quercetin-3-O-(4''-O-glucosyl)rhamnoside	4.86E+06	0.78	ND	ND
50	Flavonoids	Flavonols	-	Quercetin-3-O-(6''-acetyl)galactoside	1.18E+07	1.89	ND	ND
51	Flavonoids	Flavonols	-	Quercetin-3-O-(6''-acetyl)glucoside	4.00E+06	0.64	ND	ND
52	Flavonoids	Flavonols	-	Quercetin-3-O-(6''-malonyl)galactoside	2.37E+07	3.78	ND	ND
53	Flavonoids	Flavonols	-	Quercetin-3-O-(6''-p-Coumaroyl)galactoside	4.40E+06	0.70	ND	ND
54	Flavonoids	Flavonols	-	Quercetin-3-O-(6''-p-Coumaroyl)glucoside	3.92E+06	0.63	ND	ND
55	Flavonoids	Flavonols	22255-13-6	Quercetin-3-O-arabinoside (Guaijaverin)	1.70E+07	2.72	29.65	0.7
56	Flavonoids	Flavonols	482-36-0	Quercetin-3-O-galactoside (Hyperin)	4.92E+06	0.79	6.94	0.77
57	Flavonoids	Flavonols	482-35-9	Quercetin-3-O-glucoside (Isoquercitrin)	5.91E+06	0.94	1.86	0.77
58	Flavonoids	Flavonols	-	Quercetin-3-O-glucoside-7-O-rhamnoside	3.77E+06	0.60	ND	ND
59	Flavonoids	Flavonols	522-12-3	Quercetin-3-O-rhamnoside(Quercitrin)	5.03E+06	0.80	4.04	0.74
60	Flavonoids	Flavonols	-	Quercetin-3-O-rhamnosyl(1→2)arabinoside	5.28E+04	0.01	ND	ND

61	Flavonoids	Flavonols	52525-35-6	Quercetin-3-O-robinobioside	6.26E+06	1.00	ND	ND
62	Flavonoids	Flavonols	153-18-4	Quercetin-3-O-rutinoside (Rutin)	6.98E+05	0.11	3.2	0.68
63	Flavonoids	Flavonols	549-32-6	Quercetin-3-O-xyloside (Reynoutrin)	3.16E+07	5.06	1.68	0.7
64	Flavonoids	Flavonols	201463-36-7	Quercetin-4′-O-glucuronide	2.93E+06	0.47	ND	ND
65	Flavonoids	Flavonols	-	Quercetin-5-O-glucuronide	8.87E+06	1.42	ND	ND
66	Flavonoids	Flavonols	-	Quercetin-7-O-(6"-malonyl)glucoside	2.34E+07	3.74	ND	ND
67	Flavonoids	Flavonols	491-50-9	Quercetin-7-O-glucoside	8.20E+06	1.31	2.85	0.79
68	Flavonoids	Flavonols	571-74-4	Sexangularetin	1.23E+05	0.02	62.86	0.3
69	Flavonoids	Flavonoid carbonoside	-	Apigenin-6,8-di-C-arabinoside	2.72E+05	0.04	ND	ND
70	Flavonoids	Flavonoid carbonoside	-	Apigenin-6-C-(2"-glucosyl)arabinoside	3.60E+07	5.76	ND	ND
71	Flavonoids	Flavonoid carbonoside	-	Apigenin-6-C-(2"-rhamnosyl)glucoside	2.42E+05	0.04	ND	ND
72	Flavonoids	Flavonoid carbonoside	-	Apigenin-6-C-arabinoside-8-C-xyloside	1.27E+06	0.20	ND	ND
73	Flavonoids	Flavonoid carbonoside	29702-25-8	Apigenin-6-C-glucoside (Isovitexin)	3.62E+06	0.58	31.29	0.72
74	Flavonoids	Flavonoid carbonoside	-	Apigenin-6-C-xyloside-8-C-arabinoside	2.27E+06	0.36	ND	ND
75	Flavonoids	Flavonoid carbonoside	-	Apigenin-8-C-(2"-xylosyl)glucoside	4.04E+06	0.65	ND	ND
76	Flavonoids	Flavonoid carbonoside	3681-93-4	Apigenin-8-C-Glucoside (Vitexin)	3.70E+06	0.59	3.05	0.71

77	Flavonoids	Flavonoid carbonoside	3682-02-8	Isohemiphloin	4.53E+05	0.07	ND	ND
78	Flavonoids	Flavonoid carbonoside	-	Isovitexin-2"-O-rhamnoside	1.22E+06	0.19	ND	ND
79	Flavonoids	Flavonoid carbonoside	-	Isovitexin-8-O-xylcoside	9.31E+05	0.15	ND	ND
80	Flavonoids	Flavonoid carbonoside	29428-58-8	Luteolin-6,8-di-C-glucoside	2.19E+05	0.04	3.08	0.75
81	Flavonoids	Flavonoid carbonoside	4261-42-1	Luteolin-6-C-glucoside (Isoorientin)	1.56E+06	0.25	23.3	0.76
82	Flavonoids	Flavonoid carbonoside	28608-75-5	Luteolin-8-C-glucoside (Orientin)	5.47E+06	0.87	1.79	0.75
83	Flavonoids	Flavonoid carbonoside	-	Orientin-7-O-glucoside	2.18E+06	0.35	ND	ND
84	Flavonoids	Flavonoid carbonoside	51938-32-0	Schaftoside	1.33E+05	0.02	4.68	0.82
85	Flavonoids	Flavonoid carbonoside	61360-94-9	Vitexin-2"-O-glucoside	1.96E+06	0.31	ND	ND
86	Flavonoids	Flavonoid carbonoside	64820-99-1	Vitexin-2"-O-rhamnoside	2.41E+06	0.39	6.98	0.8
87	Flavonoids	Flavonoid	-	"Vitexin-2""-O-xyloside"	5.20E+06	0.83	ND	ND
88	Flavonoids	Flavonoid	18085-97-7	4',5,7-Trihydroxy-3',6-dimethoxyflavone (Jaceosidin)	7.80E+03	0.00	2.14	0.34
89	Flavonoids	Flavonoid	5631-70-9	5,7,4'-Trimethoxyflavone	1.04E+04	0.00	39.83	0.3
90	Flavonoids	Flavonoid	-	6-Hydroxyluteolin 5-glucoside	1.60E+07	2.57	ND	ND
91	Flavonoids	Flavonoid	480-36-4	Acacetin-7-O-rutinoside (Linarin)	1.22E+05	0.02	39.84	0.71

92	Flavonoids	Flavonoid	55167-29-8	Apiferol	4.93E+05	0.08	ND	ND
93	Flavonoids	Flavonoid	520-36-5	Apigenin	3.72E+04	0.01	23.06	0.21
94	Flavonoids	Flavonoid	23666-13-9	Apigenin-6,8-di-C-glucoside	1.40E+07	2.23	3.42	0.78
95	Flavonoids	Flavonoid	5128-44-9	Apigenin-7,4'-dimethyl ether	1.83E+04	0.00	27.12	0.27
96	Flavonoids	Flavonoid	-	Apigenin-7,4'-di-O-Glucoside	5.88E+03	0.00	ND	ND
97	Flavonoids	Flavonoid	-	Apigenin-7-O-(2"-glucosyl)arabinoside	4.22E+04	0.01	ND	ND
98	Flavonoids	Flavonoid	72741-92-5	Apigenin-7-O-(6"-acetyl)glucoside	4.17E+04	0.01	ND	ND
99	Flavonoids	Flavonoid	-	Apigenin-7-O-(6"-p-Coumaryl)glucoside	3.39E+05	0.05	ND	ND
100	Flavonoids	Flavonoid	578-74-5	Apigenin-7-O-glucoside(Cosmosiin)	3.92E+05	0.06	9.68	0.74
101	Flavonoids	Flavonoid	17306-46-6	Apigenin-7-O-neohesperidoside (Rhoifolin)	4.91E+05	0.08	6.68	0.77
102	Flavonoids	Flavonoid	552-57-8	Apigenin-7-O-rutinoside (Isorhoifolin)	4.45E+05	0.07	7.86	0.75
103	Flavonoids	Flavonoid	28189-90-4	Aromadendrin-7-O-glucoside	6.75E+05	0.11	ND	ND
104	Flavonoids	Flavonoid	-	Butin-7-O-glucoside	1.75E+06	0.28	ND	ND
105	Flavonoids	Flavonoid	301-16-6	Chrysoeriol-8-C-glucoside (Scoparin)	1.45E+05	0.02	ND	ND
106	Flavonoids	Flavonoid	520-34-3	Diosmetin (5,7,3'-Trihydroxy-4'-methoxyflavone)	7.78E+04	0.01	31.14	0.27
107	Flavonoids	Flavonoid	520-27-4	Diosmetin-7-O-rutinoside (Diosmin)	9.66E+05	0.15	12.7	0.66
108	Flavonoids	Flavonoid	22368-21-4	Eupatilin (5,7-Dihydroxy-3',4',6-Trimethoxyflavone)	3.20E+03	0.00	29.39	0.38
109	Flavonoids	Flavonoid	548-83-4	Galangin (3,5,7-Trihydroxyflavone)	6.87E+04	0.01	45.55	0.21
110	Flavonoids	Flavonoid	31712-49-9	Hesperetin-7-O-glucoside	2.27E+06	0.36	7.69	0.82
111	Flavonoids	Flavonoid	-	Kaempferol-3-O-(2"-p-Coumaroyl)galactoside	9.14E+06	1.46	ND	ND
112	Flavonoids	Flavonoid	-	Kaempferol-3-O-(3"-O-p-	7.54E+05	0.12	ND	ND

				Coumaroyl)rhamnoside				
113	Flavonoids	Flavonoid	-	Kaempferol-3-O-(4"-O-acetyl)rhamnoside	5.21E+04	0.01	ND	ND
114	Flavonoids	Flavonoid	-	Kaempferol-3-O-(4"-O-p-Coumaroyl)rhamnoside	1.05E+06	0.17	ND	ND
115	Flavonoids	Flavonoid	-	Kaempferol-3-O-(6"-galloyl)galactoside	1.30E+06	0.21	ND	ND
116	Flavonoids	Flavonoid	56317-05-6	Kaempferol-3-O-(6"-galloyl)glucoside	1.42E+06	0.23	ND	ND
117	Flavonoids	Flavonoid	-	Kaempferol-3-O-arabinoside	7.11E+06	1.14	ND	ND
118	Flavonoids	Flavonoid	-	Kaempferol-3-O-rhamnosyl(1→2)glucoside	4.15E+05	0.07	ND	ND
119	Flavonoids	Flavonoid	17650-84-9	Kaempferol-3-O-rutinoside(Nicotiflorin)	1.30E+07	2.08	3.64	0.73
120	Flavonoids	Flavonoid	19895-95-5	Kaempferol-3-O-sophoroside	2.39E+05	0.04	5.3	0.71
121	Flavonoids	Flavonoid	-	Kaempferol-4'-O-glucoside	3.08E+06	0.49	ND	ND
122	Flavonoids	Flavonoid	-	Limocitrin-3-O-galactoside	1.08E+05	0.02	ND	ND
123	Flavonoids	Flavonoid	491-70-3	Luteolin (5,7,3',4'-Tetrahydroxyflavone)	7.77E+05	0.12	36.16	0.25
124	Flavonoids	Flavonoid	5154-41-6	Luteolin-3'-O-glucoside	1.53E+05	0.02	ND	ND
125	Flavonoids	Flavonoid	6920-38-3	Luteolin-4'-O-glucoside	2.88E+06	0.46	35.94	0.79
126	Flavonoids	Flavonoid	-	Luteolin-7-O-(6"-caffeoyl)rhamnoside	5.35E+06	0.85	ND	ND
127	Flavonoids	Flavonoid	-	Luteolin-7-O-(6"-malonyl)glucoside	4.09E+04	0.01	ND	ND
128	Flavonoids	Flavonoid	5373-11-5	Luteolin-7-O-glucoside (Cynaroside)	1.62E+07	2.60	ND	ND
129	Flavonoids	Flavonoid	29741-10-4	Luteolin-7-O-glucuronide	4.12E+06	0.66	2.62	0.8
130	Flavonoids	Flavonoid	25694-72-8	Luteolin-7-O-neohesperidoside (Lonicerin)	7.02E+05	0.11	3.84	0.73
131	Flavonoids	Flavonoid	-	Naringenin-4'-O-glucoside	2.18E+06	0.35	ND	ND
132	Flavonoids	Flavonoid	520-11-6	Nepetin (5,7,3',4'-Tetrahydroxy-6-methoxyflavone)	2.14E+05	0.03	26.75	0.31

133	Flavonoids	Flavonoid	490-31-3	Robinetin	9.78E+05	0.16	6.35	0.28
134	Flavonoids	Flavonoid	20310-89-8	Saponarin(Isovitexin-7-O-glucoside)	4.96E+05	0.08	3.04	0.77
135	Flavonoids	Flavonoid	4423-37-4	Syringetin	6.44E+03	0.00	36.82	0.37
136	Flavonoids	Flavonoid	603-61-2	Tamarixetin (3,3',5,7-Tetrahydroxy-4'-Methoxyflavone)	3.40E+03	0.00	32.86	0.31
137	Flavonoids	Flavonoid	520-28-5	Tectochrysin	2.27E+03	0.00	9.57	0.2
138	Flavonoids	Flavonoid	-	Tetahydroxyflavone-7-O-glucuronide	1.88E+06	0.30	ND	ND
139	Flavonoids	Flavonoid	520-31-0	Tricetin (5,7,3',4',5'-Pentahydroxyflavone)	3.42E+06	0.55	28.87	0.28
140	Flavonoids	Flavonoid	520-32-1	Tricin (5,7,4'-Trihydroxy-3',5'-dimethoxyflavone)	2.13E+05	0.03	27.86	0.34
141	Flavonoids	Flavonoid	32769-01-0	Tricin-7-O-Glucoside	3.10E+06	0.50	5.68	0.85
142	Flavonoids	Flavonoid	-	Tricin-7-O-Glucuronide	4.74E+04	0.01	ND	ND
143	Flavonoids	Flavonoid	53766-40-8	Tricin-7-O-neohesperidoside	3.52E+04	0.01	ND	ND
144	Flavonoids	Flavanols	-	4'-Hydroxy-5,7-dimethoxyflavanone	6.34E+06	1.01	ND	ND
145	Flavonoids	Flavanols	-	5,7,3',4',5'-Pentahydroxyflavan (Tricetiflavan)	6.32E+06	1.01	ND	ND
146	Flavonoids	Flavanols	81555-08-0	8,8'-Methylenebiscatechin	4.77E+05	0.08	ND	ND
147	Flavonoids	Flavanols	154-23-4	Catechin	2.90E+06	0.46	ND	ND
148	Flavonoids	Flavanols	130405-40-2	Catechin gallate	1.33E+06	0.21	ND	ND
149	Flavonoids	Flavanols	-	Catechin-5-O-glucoside	7.45E+04	0.01	ND	ND
150	Flavonoids	Flavanols	-	Catechin-catechin-catechin	1.89E+06	0.30	ND	ND
151	Flavonoids	Flavanols	24808-04-6	Epiafzelechin	9.31E+05	0.15	ND	ND
152	Flavonoids	Flavanols	490-46-0	Epicatechin	7.24E+06	1.16	28.93	0.24
153	Flavonoids	Flavanols	1257-08-5	Epicatechin gallate	1.12E+06	0.18	ND	ND

154	Flavonoids	Flavanols	-	Epicatechin glucoside	1.11E+06	0.18	ND	ND
155	Flavonoids	Flavanols	-	Epicatechin-epiafzelechin	3.78E+05	0.06	ND	ND
156	Flavonoids	Flavanols	970-74-1	Epigallocatechin	2.46E+05	0.04	ND	ND
157	Flavonoids	Flavanols	989-51-5	Epigallocatechin-3-gallate	5.03E+06	0.80	ND	ND
158	Flavonoids	Flavanols	970-73-0	Gallocatechin	4.12E+06	0.66	ND	ND
159	Flavonoids	Flavanols	5127-64-0	Gallocatechin 3-O-gallate	6.43E+05	0.10	ND	ND
160	Flavonoids	Dihydroflavonol	52117-69-8	3-O-Acetylpinobanksin	1.34E+06	0.21	ND	ND
161	Flavonoids	Dihydroflavonol	480-20-6	Aromadendrin (Dihydrokaempferol)	6.88E+05	0.11	24.15	0.24
162	Flavonoids	Dihydroflavonol	27200-12-0	Dihydromyricetin (Ampelopsin)	2.37E+05	0.04	ND	ND
163	Flavonoids	Dihydroflavonol	480-18-2	Dihydroquercetin(Taxifolin)	2.13E+06	0.34	57.84	0.27
164	Flavonoids	Dihydroflavonol	20725-03-5	Fustin	1.05E+04	0.00	ND	ND
165	Flavonoids	Dihydroflavonol	548-82-3	Pinobanksin	2.02E+05	0.03	ND	ND
166	Flavonoids	Dihydroflavone	-	5,7,3',4',5'-Pentahydroxydihydroflavone	1.77E+06	0.28	ND	ND
167	Flavonoids	Dihydroflavone	492-14-8	Butin	1.32E+05	0.02	ND	ND
168	Flavonoids	Dihydroflavone	552-58-9	Eriodictyol (5,7,3',4'-Tetrahydroxyflavanone)	5.81E+05	0.09	ND	ND
169	Flavonoids	Dihydroflavone	-	Eriodictyol-7-O-(6"-malonyl)glucoside	8.09E+05	0.13	ND	ND
170	Flavonoids	Dihydroflavone	480-41-1	Naringenin (5,7,4'-Trihydroxyflavanone)	6.12E+05	0.10	42.36	0.21
171	Flavonoids	Dihydroflavone	529-55-5	Naringenin-7-O-glucoside (Prunin)	2.24E+06	0.36	9.33	0.74
172	Flavonoids	Dihydroflavone	10236-47-2	Naringenin-7-O-Neohesperidoside(Naringin)	1.35E+05	0.02	ND	ND
173	Flavonoids	Dihydroflavone	14259-46-2	Naringenin-7-O-Rutinoside(Narirutin)	3.27E+04	0.01	ND	ND
174	Flavonoids	Chalcones	14917-41-0	3,4,2',4',6'-Pentahydroxychalcone	2.63E+04	0.00	ND	ND
175	Flavonoids	Chalcones	-	Dihydrocharcone-4'-O-glucoside	6.57E+06	1.05	ND	ND
176	Flavonoids	Chalcones	4547-85-7	Isosalipurposide (Phlorizin Chalcone)	5.23E+05	0.08	ND	ND

177	Flavonoids	Chalcones	73692-50-9	Naringenin chalcone	1.91E+05	0.03	ND	ND
178	Flavonoids	Chalcones	60-82-2	Phloretin	3.42E+05	0.05	ND	ND
179	Flavonoids	Chalcones	-	Phloretin-2'-O-(6"-O-xylosyl)glucoside	8.85E+04	0.01	ND	ND
180	Flavonoids	Chalcones	60-81-1	Phloretin-2'-O-glucoside (Phlorizin)	3.87E+06	0.62	2.88	0.6
181	Flavonoids	Chalcones	4192-90-9	Phloretin-4'-O-glucoside (Trilobatin)	2.03E+06	0.32	ND	ND
182	Flavonoids	Anthocyanins	47705-70-4	Cyanidin-3-O-glucoside (Kuromanin)	3.24E+06	0.52	ND	ND
183	Tannins	Tannin	18483-17-5	1,3,6-Tri-O-galloyl-D-glucose	5.05E+06	0.81	3.01	0.54
184	Tannins	Tannin	-	1,6-Di-O-Galloyl-D-Glucose	2.07E+06	0.33	ND	ND
185	Tannins	Tannin	58511-73-2	1-O-Galloyl-D-glucose	1.36E+05	0.02	ND	ND
186	Tannins	Tannin	-	1-O-Galloyl-rhamnose	5.71E+03	0.00	ND	ND
187	Tannins	Tannin	88038-12-4	Cinnamtannin B2	4.47E+05	0.07	ND	ND
188	Tannins	Tannin	76250-49-2	Gambiriin A1	2.34E+05	0.04	ND	ND
189	Tannins	Proanthocyanidins	41743-41-3	Procyanidin A2	1.38E+05	0.02	12.25	0.4
190	Tannins	Proanthocyanidins	20315-25-7	Procyanidin B2	1.92E+06	0.31	67.87	0.66
191	Tannins	Proanthocyanidins	37064-30-5	Procyanidin C1	7.52E+05	0.12	18.98	0.1

OB, oral bioavailability; DL, drug-likeness; NI: no information has been found.
