# Supplementary material for: Hawk Tea Flavonoids as Natural Hepatoprotective Agents Alleviate Acute Liver Damage by Reshaping the Intestinal Microbiota and Modulating the Nrf2 and NF-κB Signaling Pathways
Source: Nutrients. 2022 Sep 5;14(17):3662. doi: 10.3390/nu14173662 (PMC9459715; doi:10.3390/nu14173662)
Supplement: Supplementary file 1 [file nutrients-14-03662-s001.zip › nutrients-1875575-supple/Table. S2.pdf]

Table S2. The Product Codes of Antibodies and ELISA Kit

Antibodies	Product Codes	Source	Dilution ratio
TLR4	66350-1-Ig	Proteintech Group, Inc.	1:4000
MyD88	67969-1-Ig	Proteintech Group, Inc.	1:5000
NRF2	16396-1-AP	Proteintech Group, Inc.	1:3000
HO-1	66743-1-Ig	Proteintech Group, Inc.	1:3000
NF-kappaB	80979-1-RR	Proteintech Group, Inc.	1:20000
GAPDH	10494-1-AP	Proteintech Group, Inc.	1:20000

ELISA Kit	Product Codes	Source
IL-6	H007-1-1	Nanjing Jiancheng Bioengineering Institute
IL-1β	H002	Nanjing Jiancheng Bioengineering Institute
TNF-α	H052-1	Nanjing Jiancheng Bioengineering Institute

**Observed molecular weight**
96 kDa
33 kDa
110 kDa
33 kDa
65 kDa
36 kDa
