# Supplementary figures and images for: Hawk Tea Flavonoids as Natural Hepatoprotective Agents Alleviate Acute Liver Damage by Reshaping the Intestinal Microbiota and Modulating the Nrf2 and NF-κB Signaling Pathways
Source: Nutrients. 2022 Sep 5;14(17):3662. doi: 10.3390/nu14173662 (PMC9459715; doi:10.3390/nu14173662)

Figure S1

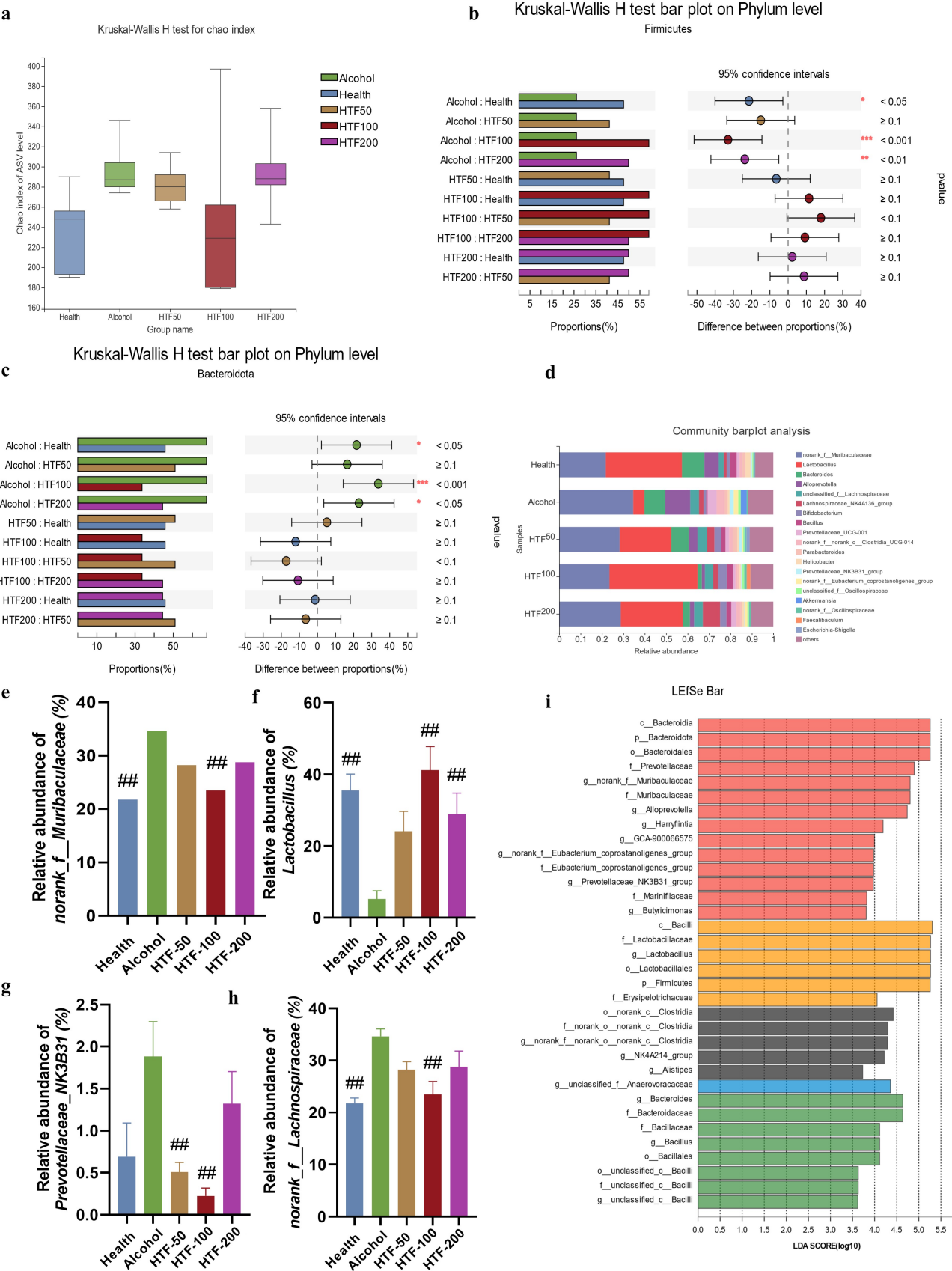

Supplement: Supplementary file 1 [file nutrients-14-03662-s001.zip › nutrients-1875575-supple/Fig.S1.pdf]
